# ﻿A new species, *Didymoglossumradiatum* (Hymenophyllaceae), and two new records with note to the genus *Didymoglossum* Desv. from Thailand

**DOI:** 10.3897/phytokeys.261.157609

**Published:** 2025-08-06

**Authors:** Siwakorn Chokrassameehirun, Ekaphan Kraichak, Tassanai Jaruwattanaphan

**Affiliations:** 1 Department of Horticulture, Faculty of Agriculture, Kasetsart University, Bangkok 10900, Thailand Kasetsart University Bangkok Thailand; 2 Department of Botany, Faculty of Science, Kasetsart University, Bangkok 10900, Thailand Kasetsart University Bangkok Thailand; 3 Biodiversity Center Kasetsart University, Bangkok 10900, Thailand Kasetsart University Bangkok Thailand

**Keywords:** Diversity, filmy ferns, *Microgonium*, peltate frond, *Trichomanes*

## Abstract

During the filmy fern (Hymenophyllaceae) diversity study in Thailand, the genus *Didymoglossum* was investigated using morphological data and molecular phylogenetic analyses of the chloroplast *rbcL* region. This study revealed a new species, *Didymoglossumradiatum* S.Chokrassameehirun, Kraichak & Jaruwatt., **sp. nov.**, which is currently known only from peninsular Thailand. This taxon resembles *D.tahitense* in possessing peltate fronds but differs in frond size, the structure of false veinlets, and the appearance of its sori. The phylogenetic results further confirmed the distinct phylogenetic positions of *D.henzaianum* and *D.mindorense*, which were also newly recorded and described for Thailand.

## ﻿Introduction

The genus *Didymoglossum* Desv. (sensu Ebihara et al. 2006) is a group of dwarf epiphytic or epilithic ferns in the family Hymenophyllaceae. Currently, it comprises approximately 30 species, primarily distributed in tropical regions (Dubuisson et al. 2023). Of these, fifteen species have been recorded in the Old World, while only eight occur in tropical Asia (Ebihara et al. 2006; Senterre et al. 2017). Phylogenetic analyses based on plastid DNA (*rbcL*) and morphological characters support the division of this genus into two subgenera (Dubuisson et al. 2003, 2023; Ebihara et al. 2006, 2007; Senterre et al. 2017). The subgenus Microgonium (C.Presl) Ebihara & Iwats. is usually distinguished by the presence of submarginal false veinlets and is found in the Paleotropics. In contrast, submarginal false veinlets are typically absent in subg. Didymoglossum, which is more abundant in the Neotropics (Copeland 1933, 1938; Croxall 1985; Ebihara et al. 2006; Senterre et al. 2017; Dubuisson et al. 2023).

In Asia, five species have been recorded from India (Krishnan and Rekha 2021) and China (Liu et al. 2013), along with a newly recorded species from Vietnam (Chen et al. 2020). Additionally, three species and one unidentified taxon of *Didymoglossum* were reported from the Solomon Islands and Seram by Chen et al. (2017, 2022) and Iwatsuki et al. (2019). A recent study by Iwatsuki and Ebihara (2023) documented six species in Malesia and the surrounding regions. While many of these taxa are possibly present in Thailand, the lack of voucher specimens and dedicated researchers has left this issue unresolved.

According to previous studies on pteridophytes in Thailand (Tagawa and Iwatsuki 1979; Boonkerd and Pollawatn 2000; Lindsay et al. 2009), only four species of *Didymoglossum* have been described in the country: *D.bimarginatum* (Bosch) Ebihara & K.Iwats., *D.exiguum* (Bedd.) Copel., *D.motleyi* (Bosch) Ebihara & K.Iwats., and *D.sublimbatum* (Müll. Berol.) Ebihara & K.Iwats. However, no additional species from this genus have been reported until now. During the revision of Hymenophyllaceae in Thailand, numerous intriguing specimens were collected and examined. Through molecular phylogenetic analysis and morphological investigations, we describe one new species and report two newly recorded species of *Didymoglossum* from Thailand. An updated key to the Thai species of *Didymoglossum* is also provided.

## ﻿Materials and methods

### ﻿Specimen examination and fieldwork

This study was based on field collections from 2023–2024 and specimens examined in Thailand based on BCU, BK, BKF, CMU, CMUB, PSU, and QBG (herbarium acronyms followed Thiers 2024, continuously updated). Digital images of type and voucher specimens were accessed through the Pteridophyte Portal Consortium (PCC) data portal (Rothfels et al. 2025). The classification system follows Ebihara et al. (2006).

### ﻿Taxon sampling

Seven species of *Didymoglossum* were included in the molecular analyses, along with three species from the genera *Crepidomanes* C. Presl and *Hymenophyllum* Sm., selected as outgroup taxa. Total genomic DNA was extracted using the DNeasy Plant Pro Kit® (Qiagen®, Germany), following the manufacturer’s protocol. The plastid *rbcL* gene was chosen for phylogenetic analyses due to the availability of comparative data and its widespread usage within *Trichomanes* s.l. studies. The primers *rbcL*-TKT-F1 (5’–ACCCAWGTCACCACAAACRGAG–3’) and *rbcL*-TKT-R3N-2 (5’–CAAGCGGCAGCCRAYTCAG–3’) were used for *rbcL* amplification (Ebihara et al. 2007). Amplification was carried out in a 25 µl reaction using Quick Taq™ HS DyeMix (Toyobo Inc., Japan) for 35 cycles under the following conditions: 95 °C (15 sec), 53 °C (15 sec), and 72 °C (90 sec), with pre-denaturation at 95 °C (1 min) and final extension at 72 °C (10 min). The resulting products were purified and sequenced by Macrogen, Inc. (Korea).

### ﻿Phylogenetic analyses

A total of 48 *rbcL* sequences from GenBank, including 14 newly generated sequences from Thailand (GenBank accession numbers for all sequences are provided in Appendix 1), were analyzed. All sequences were aligned using MUSCLE (Edgar 2004) in MEGA 11 (Tamura et al. 2021). The nucleotide substitution model for the alignment was selected based on the AICc criteria via MrModel (Darriba et al. 2019) in the CIPRES gateway (Miller et al. 2011). The best-fit model, GTR+I+G4, was set for maximum likelihood (ML) and Bayesian inference (BI) phylogenetic analyses. The phylogenetic analyses were performed in the CIPRES gateway using IQ-TREE v. 2.4.0 (Minh et al. 2020) and MrBayes v. 3.2.7a (Ronquist et al. 2012), respectively. For ML phylogeny, 1000 bootstrap replicates were performed to assess bootstrap support (BS). BI analysis was conducted with two simultaneous runs, each consisting of four chains. A total of three million generations were executed, with trees sampled every 1,000 generations. The first 25% of generations were discarded as burn-in, and posterior probability (PP) support values were summarized.

## ﻿Results

**Figure 1.**
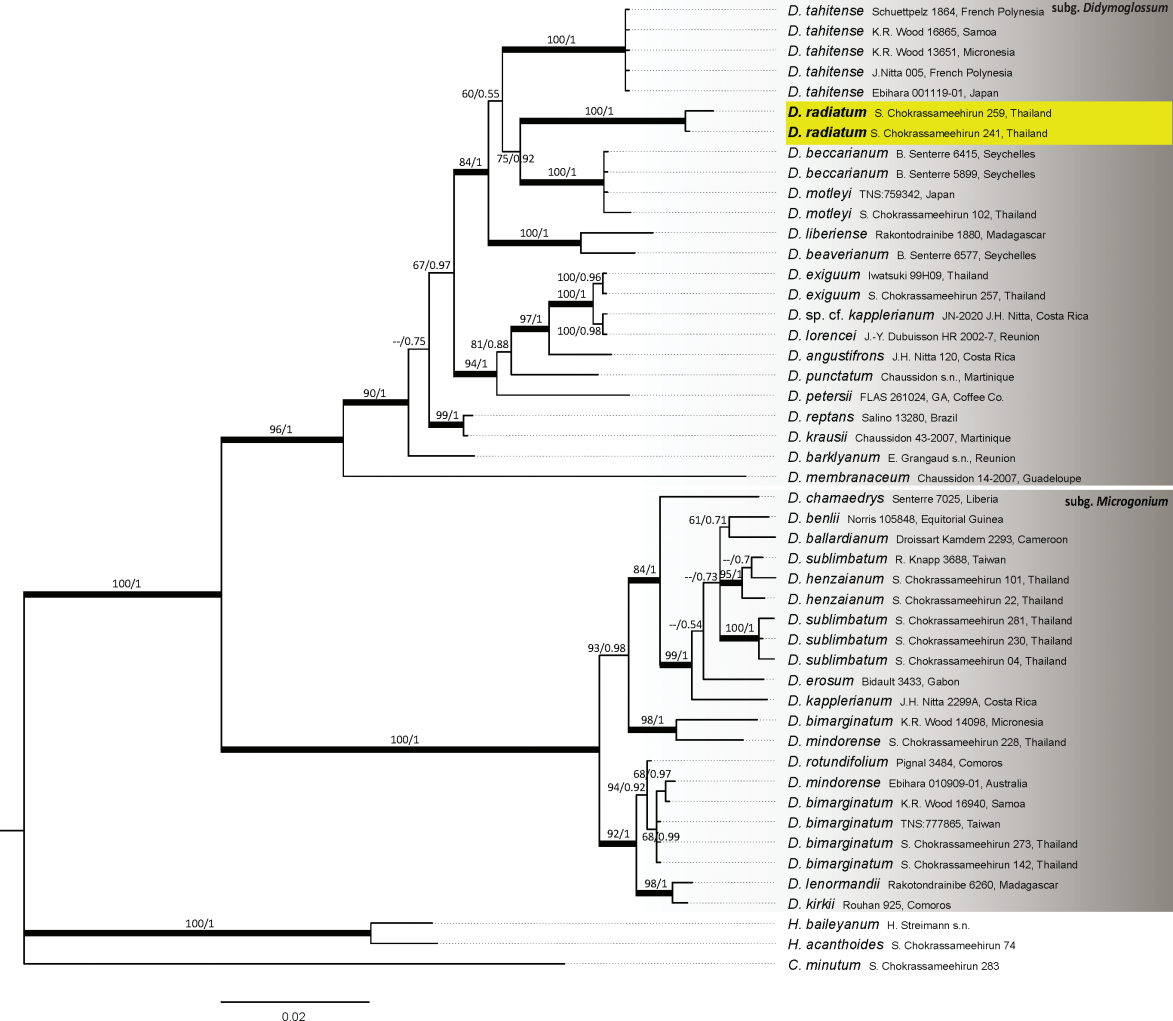
Bayesian phylogenetic analysis (BI) phylogeny of *Didymoglossum* based on chloroplast *rbc*L (1206 bp) sequences. Branch support indicated ML bootstrap/BI posterior probabilities value. “--” indicated an ML bootstrap value less than 60. The bold line indicated BI posterior probabilities = 1. Highlighted showing the phylogenetic position of the new species described in the text.

The final alignment of *rbcL* sequences was 1206 bp with 338 informative sites. Topologies of phylogenetic trees derived from maximum likelihood and Bayesian inference methods exhibited no conflicts (Fig. 1). The monophyly of the two subgenera within *Didymoglossum*, subg. Didymoglossum and subg. Microgonium, was supported (BS/PP = 96/1 and 100/1, respectively). The newly described species clearly belongs to subg. Didymoglossum. It is sister to a clade comprising *D.beccarianum* (Ces.) Senterre & Rouhan and *D.motleyi* (100/1), with moderate to strong support (75/0.92). Notably, its frond morphology strongly resembles *D.tahitense* (Nadeaud) Ebihara & K.Iwats., which is genetically distinct. Within subg. Microgonium, the Thai samples of *D.sublimbatum* form a strongly supported clade (100/1), whereas *D.sublimbatum* from Taiwan is nested within *D.henzaianum* (Parish ex Hook.) Mazumdar from Thailand (95/1), possibly due to misidentification of the Taiwanese *D.sublimbatum* specimens, as these two species are morphologically similar. *Didymoglossummindorense* (Christ) K.Iwats. from Thailand was closely related to *D.bimarginatum* from Micronesia (98/1), but other *D.bimarginatum* samples formed a separate clade (68/0.99), including one sample of *D.mindorense* (Ebihara 010909-01, Australia). The conflicting specimens, *D.bimarginatum* from Micronesia and *D.mindorense* from Australia, appear to be misidentified, likely representing a situation similar to that of *D.sublimbatum* from Taiwan.

### ﻿Taxonomic treatment

#### ﻿ Didymoglossum radiatum

Taxon classificationPlantaeHymenophyllalesHymenophyllaceae

S.Chokrassameehirun, Kraichak & Jaruwatt. sp. nov.

E277DE2D-77A6-5015-8C43-F9DD334C90DD

urn:lsid:ipni.org:names:77366765-1

Figs 2 , 3 , 6A–C

##### Type.

Thailand • Yala: Waeng District, Hala–Bala Wildlife Sanctuary, 5°47'N, 101°49'E, c. 112 msl., 25 Mar. 2024, *S. Chokrassameehirun 24-259* (holotype: BKF; isotype: QBG)

##### Diagnosis.

*Didymoglossumradiatum* is similar to *D.tahitense* (Fig. 6D–F) and *D.hildebrandtii* (Kuhn) Ebihara & Dubuisson in having peltate fronds but differs in several key characters. In *D.tahitense* and *D.hildebrandtii*, the false veinlets are branched, forming dichotomous branching in almost all veins (Fig. 6E). In contrast, the false veinlets of *D.radiatum* are parallel, unconnected from the inner base of the false veins throughout their entire length. The frond diameter of *D.radiatum* is smaller (c. 1.5 cm) compared to *D.tahitense* (c. 1–3 mm) (Fig. 6D). The sori of *D.radiatum* are raised, solitary, c. 2 mm long, usually sunk in the deep notch of the fronds. In comparison, the sori of *D.tahitense* can reach up to 4 mm in length and are situated in shallow marginal notches (Fig. 6F), whereas *D.hildebrandtii* has multiple sori per frond. Additionally, *D.hildebrandtii* is distributed in the Comoros and East Africa, unlike *D.tahitense*, which occurs sympatrically with *D.radiatum* in the Malesian region. A closely related species, *D.beccarianum*, is readily distinguished by its stipitate fronds, pinnate arrangement of false veinlets, and sori in the apical notch of fertile fronds.

##### Description.

***Rhizomes***: long-creeping, slender, c. 0.2–0.3 mm in diameter, densely covered with dark brown hairs, tightly attached to substrate. ***Fronds***: sessile, peltate, c. 1–1.5 cm in diameter, circular to elliptic, margin of fronds densely covered with short dark brown hairs. ***False veinlets***: dense, connected to joint of fronds, parallel and not connected to each other, running straight to margin, obsolete near apex, densely covered with dark brown hairs underside, and tightly attached to substrate. ***Sori***: solitary, usually one per frond, sunk in deeply notch, borne on main veins running straight from joint of frond, raised, ascending to erect, anticlinal to substrate. ***Involucres***: tubular, c. 2–2.5 mm long, fully emerging from fronds, wings very narrow, forming ridge in appearance, broadly dilated at apex. ***Receptacles***: filiform long exerted.

**Figure 2.**
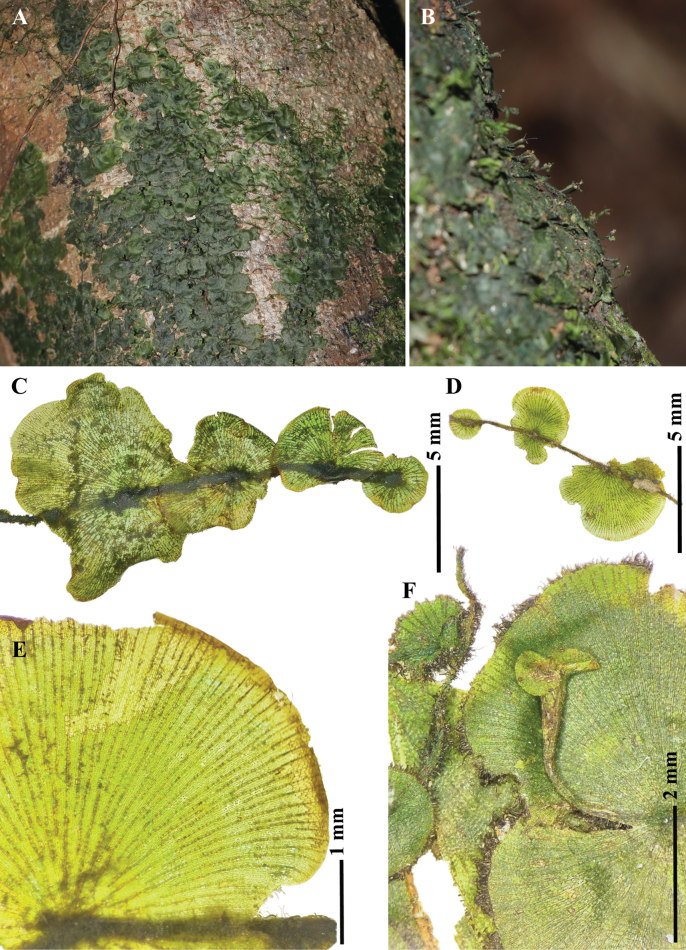
*Didymoglossumradiatum* S.Chokrassameehirun, Kraichak & Jaruwatt. from the holotype *S. Chokrassameehirun 24-259* (BKF) A. Habit; B. Raised sori of fertile plants; C. Adaxial surface of sterile fronds; D. Abaxial surface of sterile fronds; E. False veinlet construction; F. Sori. Photographed by S. Chokrassameehirun.

##### Distribution and habitat.

This species is only known from Thailand, adjacent to Malaysia. Sparse populations were discovered near the type locality in a nationally conserved area. Epiphytic or epilithic habit, usually near the streamlet at low elevations of lowland evergreen forest.

**Figure 3.**
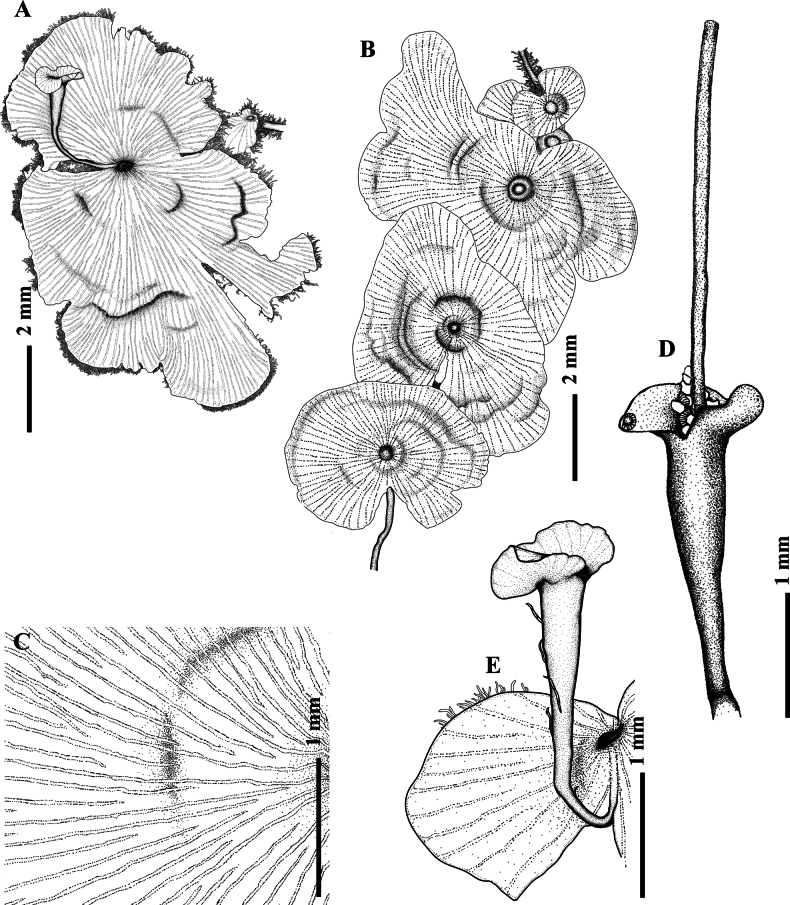
Illustration of *Didymoglossumradiatum* S.Chokrassameehirun, Kraichak & Jaruwatt. based on the holotype *S. Chokrassameehirun 24-259* (BKF). A. Fertile frond; B. Sterile fronds; C. False veinlet construction; D–E. Sori with tubular involucres; D. Sori with filiform receptacle; E. Sori borne at the notch of the frond. Illustrated by S. Chokrassameehirun.

##### Etymology.

The specific epithet –*radiatum* refers to the radiate and parallel arrangement of false veinlets on the fronds.

##### Vernacular name.

The Thai name “Fern Bai Bang Rat Sa Mi” (เฟินใบบางรัศมี) relates to the specific epithet.

##### Additional specimens examined.

Thailand • Yala: Waeng District, Hala–Bala Wildlife Sanctuary, Ai Ka Ding canal, c. 190 msl., 24 Mar. 2024, *S. Chokrassameehirun 24-241* (BKF).

###### ﻿New records for Thailand

#### ﻿ Didymoglossum henzaianum

Taxon classificationPlantaeHymenophyllalesHymenophyllaceae

(Parish ex Hook.) Mazumdar, Phytotaxa 158: 297, 2014.

B74CF5F9-FD20-5459-8278-C7F6882AC5E8

Figs 4 , 6G–I

##### Type.

Myanmar • Mawlamyine, Henzai basin, *C.S.P. Parish 5* (holotype: K [K000974336]; isotype: BM [BM001073866, photo seen], GH).

##### Diagnosis.

This species is frequently misidentified as *D.sublimbatum* (Fig. 6J–L), but *D.henzaianum* has notably smaller fronds, typically about 1.5 cm long. The sterile fronds are round or linear, while fertile fronds range from ovate to spatulate. In contrast, *D.sublimbatum* has larger fronds (usually 2–3 cm long) that are oblong to lanceolate.

##### Description.

***Rhizomes***: long, creeping, slender, densely covered with short brown hairs. ***Stipes***: very short, less than 2 mm long, terete in basal portion, sparsely covered with dark brown multicellular hairs. ***Fronds***: entire to slightly lobed, with various shapes, round to ovate or linear to spatulate, 0.5–2 cm long, 0.5 cm broad, moderately acute to round at apex, cuneate at base, margin entire or slightly undulate. ***False veinlets***: many, long, some false veins connected to main veins, usually 5–8 cells between adjacent false veins, apex of false veins usually falcate, forming sub-parallel to margin and connected to upper ones but not continuous. ***Sori***: solitary, sunk in the apical segment, usually 1–2 sori per frond. ***Involucres***: obconic, broadly dilated at the mount of involucres. ***Receptacles***: filiform, long exserted.

**Figure 4.**
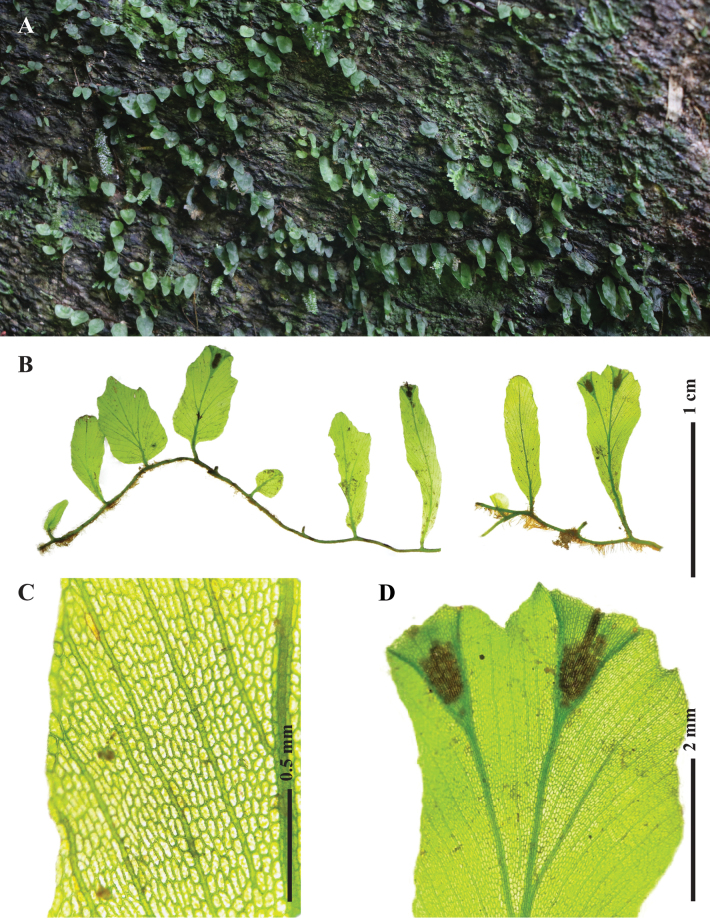
*Didymoglossumhenzaianum* (Parish ex Hook.) Mazumdar from the voucher *S. Chokrassameehirun 23-105* (BKF). A. Habit; B. Fertile plants; C. False veinlets on lamina segment; D. Sori with obconic–tubular involucres. Photographed by S. Chokrassameehirun.

##### Distribution and habitat.

India, Myanmar, Vietnam, Thailand, and Malesia (Peninsular Malaysia). Epilithic near streams.

##### Additional specimens examined.

Thailand • Loei: Phu Ruea District, 31 Aug. 2020, *S. Chokrassameehirun 20-22* (BKF); Kanchanaburi: “Klawng Wa,” 24 Dec. 1928, *A.F.G. Kerr 16321* (BK); Surat Thani: Phanom District, 16 Oct. 2023, *S. Chokrassameehirun 24-105* (BKF).

#### ﻿ Didymoglossum mindorense

Taxon classificationPlantaeHymenophyllalesHymenophyllaceae

(Christ) K.Iwats. in Ebihara et al., Blumea 51: 236, 2006.

49912CD1-587F-5AD0-BD5C-D2FDF6DC1A86

Figs 5 , 6M–O

##### Type.

Philippines • Mindoro, *E.D. Merrill 6066* (isolectotype, designated by Croxall, Austral. J. Bot. 23(2): 538 (1975), GH [GH00022253, photo seen], MICH [MICH1191083, photo seen], P [P00624463, photo seen], US [US00134614, photo seen]).

##### Diagnosis.

*Didymoglossummindorense* resembles *D.bimarginatum* (Fig. 6P–R) but differs in having deeply lobed segments and fewer false veinlets, which are more widely spaced.

##### Description.

***Rhizomes***: filiform, long-creeping, densely covered with dark brown hairs. ***Stipes***: short, to 5 mm long. ***Fronds***: less than 3 cm long, usually 2–2.5 cm long, c. 1.2 cm broad, entire to pinnately lobed, round at apex, attenuate to broadly cuneate at base. ***Costa***: glabrous, obsolete near apex. ***Submarginal false veinlets***: continuous with 1–2 rows of normal cells outside. ***False veinlets***: long, distantly placed, connecting and running from main veins to submarginal ones, distancely placed, usually 6–10 cells between adjacent false veins. ***Lobes***: obtuse at apex, usually 0.5–0.7(–1) cm long, slightly undulate. ***Sori***: solitary, sunk in lobes. ***Involucres***: tubular, with a distinctly dilated mouth. ***Receptacles***: long exserted, broken when aged.

**Figure 5.**
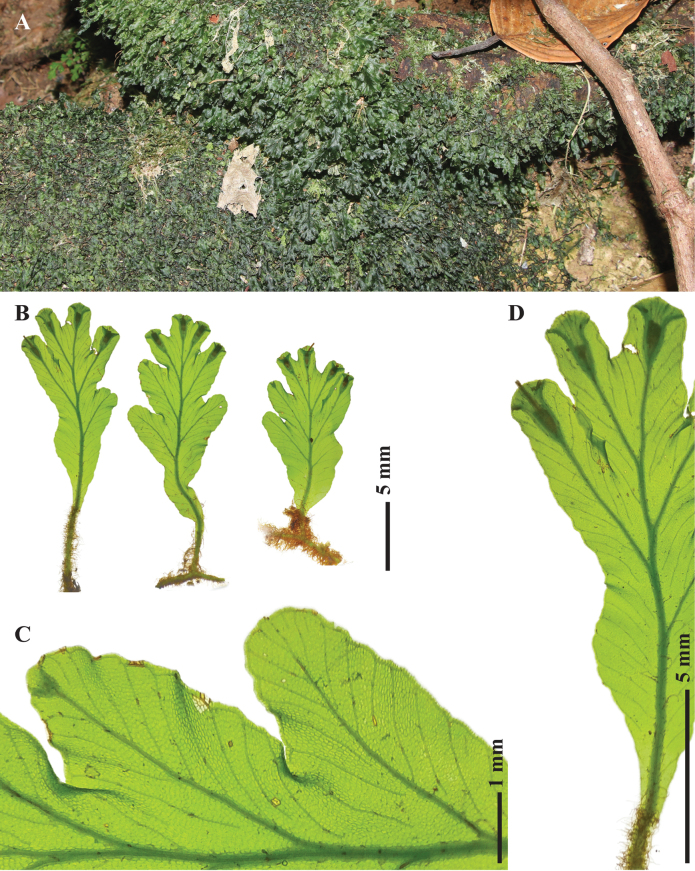
*Didymoglossummindorense* (Christ) K.Iwats. from the voucher *S. Chokrassameehirun 24-228* (BKF). A. Habit; B. Fertile fronds; C. Lobe of sterile frond; D. Fertile frond. Photographed by S. Chokrassameehirun.

##### Distribution and habitat.

Thailand, Malesia (Borneo, Philippines, New Guinea), Solomon Islands, Australia (NE Queensland). Epiphyte or epilithic near streams at lower elevations, usually growing at the base of the tree.

**Figure 6.**
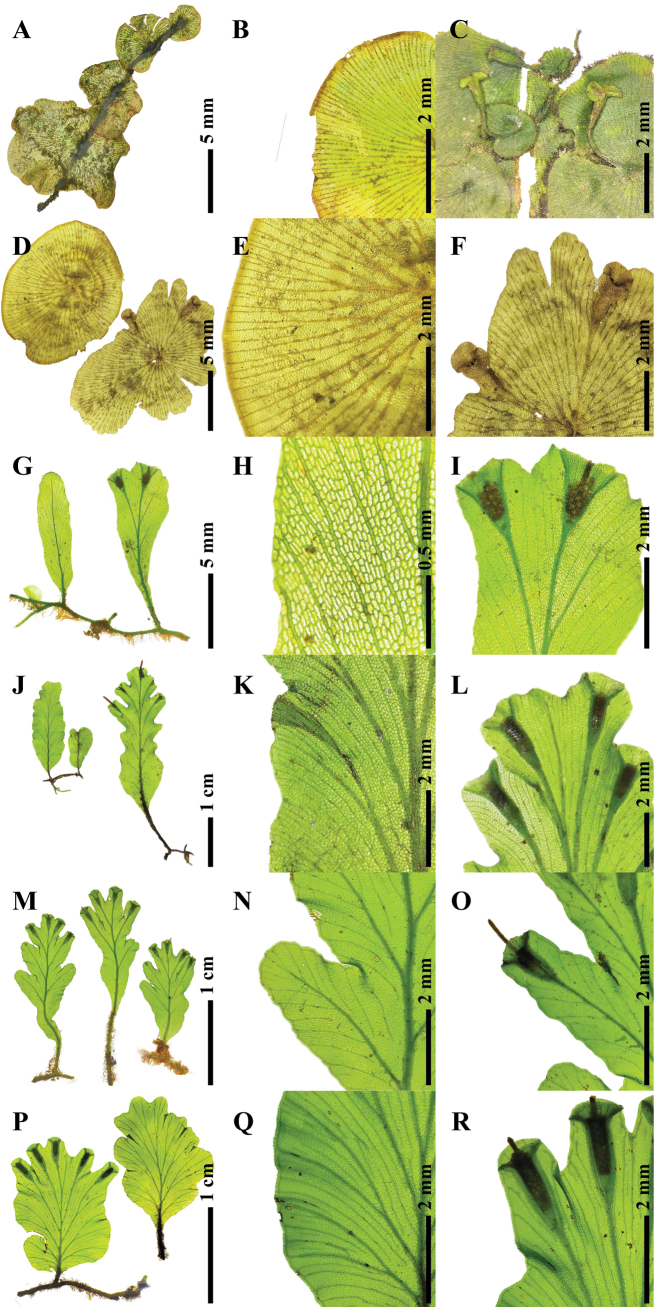
Morphological comparison of some *Didymoglossum* species. A–C. *D.radiatum*; D–F. *D.tahitense*; G–I. *D.henzaianum*; J–L. *D.sublimbatum*; M–O. *D.mindorense*; P–R. *D.bimarginatum*; A, D, G, J, M, P. Fronds morphology; B, E, H, K, N, Q. Laminae segments showing false veinlet characters; C, F, I, L, O, R. Sori position and morphology. Photographed by S. Chokrassameehirun.

##### Additional specimens examined.

Thailand • Yala: Ban Chulapjon Phatthanna 7, 13 Jun. 2004, *S. Saengrit 019* (BKF); • ibid., 15 Jul. 2004, *S. Saengrit 020* (BKF); Narathiwat: Ta Mo, 10 Jun. 2004, *S. Saengrit 016* (BKF); Hala–Bala Wildlife Sanctuary, 22 Jul. 2004, *S. Saengrit 028* (BKF); • ibid., 9 Jun. 2004, *S. Saengrit 009* (BKF); • ibid., 23 Mar. 2024, *S. Chokrassameehirun 24-228* (BKF); • ibid., 24 Mar. 2024, *S. Chokrassameehirun 24-236* (BKF); *ibid.*, 25 Mar. 2024, *S. Chokrassameehirun 24-256* (BKF).

## ﻿Discussion

Consistent with the recent classification of *Didymoglossum* (Ebihara et al. 2006), our phylogenetic analysis supports the recognition of two subgenera within the genus. The phylogeny placed the new species, *D.radiatum*, in subg. Didymoglossum, consistent with its lack of submarginal false veinlets. The new species is closely related to a clade including *D.beccarianum* and newly sequenced material of *D.motleyi* from Thailand. This clade was previously described by Senterre et al. (2017), who treated all of its members as *D.beccarianum*; this treatment was later adopted in recent fern studies in Taiwan (TPG-Taiwan Pteridophyte Group 2019) and the Solomon Islands (Chen et al. 2017, 2022). *Didymoglossummotleyi* has been referenced in various floristic studies (Liu et al. 2013; Iwatsuki and Ebihara 2023), including in Thailand, where Tagawa and Iwatsuki (1979) documented this species for the Flora of Thailand based on frond morphology and sori characters. Our phylogeny and morphological data indicate that *D.motleyi* in Thailand may be a synonym of *D.beccarianum*, in agreement with the treatment of Senterre et al. (2017) restricting the distribution of *D.motleyi* to its type locality (Borneo). However, no DNA sequence of *D.motleyi* from the type locality is available, and broader sampling and more comprehensive studies are needed to clarify the status of the *D.motleyi* complex (Senterre et al. 2017).

*Didymoglossumexiguum* is morphologically distinctive, with marginal hairs being a key characteristic of this taxon. Our results confirm its monophyly, though based on only two samples from Thailand. This species, however, has a broader distribution, extending across Sri Lanka, India, Thailand, Peninsular Malaysia, and Australia (Iwatsuki and Ebihara 2023). Liu et al. (2013) and Chen et al. (2020) recorded a similar species, *D.wallii* (Thwaites) Copel., from China and Vietnam. These species share morphological similarities, including the presence of black marginal hairs, frond shape and size, and a tubular indusium with dilated, bicolored lips. The type specimens of both species were collected from adjacent areas: *D.exiguum* from South India (Beddome s.n., lectotype: K [K001090183, photo seen], isolectotype: BM) and *D.wallii* from “Ceylon” (Sri Lanka) (Wall s.n., holotype: K [K001090167, photo seen], isotype: LE [LE00007961]). Copeland (1933) provided a detailed comparison of these species, primarily distinguishing them by the position of the involucre (completely immersed in *D.wallii* vs. exserted in *D.exiguum*) and the density of false veinlets (fewer in *D.wallii* vs. more numerous in *D.exiguum*). While these traits exhibit some variation, false veinlet density is likely a distinguishing trait of *D.wallii*. Future studies with broader sampling and morphological analyses are needed to clarify the distinction between these species.

Within Thailand, *D.sublimbatum* forms a monophyletic clade distinct from specimens from Taiwan, which are nested within *D.henzaianum*. Closer examination of digital images of the problematic Taiwanese specimens (Knapp 3688, P [P01189828, photo seen]) suggests that their small (c. 2 cm) fertile fronds, obconic sori, and spatulate frond shape are characteristic of *D.henzaianum*, which suggests misidentification of this specimen. In contrast, *D.sublimbatum* is more widely recorded across various regions in Thailand, with its morphology aligning with the type specimens (Zollinger 865, lectotype: B [B200106841, photo seen], isolectotype: K [K000375036, photo seen], MEL, P). The allied species, *D.henzaianum*, was initially identified as *D.sublimbatum* in the *Flora of Thailand* (Tagawa and Iwatsuki 1979). Croxall (1985) mentioned only the voucher specimen collected by Kerr 16874 (K) from Thailand, without formally describing Thai material. After a careful investigation of a duplicate specimen at BK (BK253137!), we concur with Croxall (1985) and confirm the presence of this species in Thailand. Phylogenetically, *D.sublimbatum* and *D.henzaianum* are nested within subgenus Microgonium, which conflicts with the traditional morphological concept of the subgenus. Both species lack submarginal false veinlets, a key characteristic of subgenus Didymoglossum. This phylogenetic conflict was previously noted in studies of *Didymoglossum* from Africa (Dubuisson et al. 2023) and the *D.motleyi* complex (Senterre et al. 2017). The inclusion of Thai specimens in our study reinforces the need to reassess the infrageneric classification of this genus.

The Thai specimen of *D.mindorense* formed a clade with *D.bimarginatum* from Micronesia (Wood 14098 [PTBG1000019018, photo seen]). As described by Copeland (1933), *D.bimarginatum* has numerous false veinlets and laminae that are not distinctly lobed, whereas *D.mindorense* has fewer false veinlets and distinctly lobed laminae. The two specimens share key characters of *D.mindorense*, such as distinctly lobed laminae, but differ slightly in frond morphology. The Micronesian specimen identified as *D.bimarginatum* has fewer and narrower segments compared to the Thai *D.mindorense*, which more closely resembles the type specimen. In combination with the high genetic distances between these specimens, these results suggest that *D.bimarginatum* from Micronesia is probably an undescribed species. The type specimen of *D.mindorense* from Mindoro, Philippines (Merrill 6066, lectotype: P [P00624464, photo seen], isolectotype: GH, K, MICH [MICH1191083, photo seen], US) morphologically corresponds to the Thai *D.mindorense*, and the species is primarily distributed across Southeast Asia (Iwatsuki and Ebihara 2023).

The remaining *D.bimarginatum* specimens from Samoa, Taiwan, and Thailand formed a weakly supported clade together with *D.mindorense* from Australia. This clade is in turn sister to *D.rotundifolium*, which is morphologically distinct, characterized by mostly solitary sori per fertile frond, and is distributed in the Comoros and Madagascar (type locality, H. Perrier de la Bâthie 7735, syntype: P [P00482612, photo seen]; isosyntype: P [P00482613, P00477780, photo seen]). *Didymoglossumbimarginatum* typically has many sori per fertile frond and is mainly distributed in southern to southeastern Asia. The Thai *D.bimarginatum* specimens are very similar to the type in morphology. Therefore, the inclusion of the Australian specimen identified as *D.mindorense* in the *D.bimarginatum* clade is questionable, but pending voucher specimens for further investigation, we assume this is probably a misidentified sample that needs re-examination.

### ﻿Key to the species of *Didymoglossum* Desv. in Thailand

**Table:** 

1	Submarginal false veinlet present	**6**
–	Submarginal false veinlet absent	**2**
2	Marginal hairs present	***D.exiguum***
–	Marginal hairs absent	**3**
3	Fronds sessile, peltate, orbiculate	***D.radiatum***
–	Fronds stipitate	**4**
4	Fronds rounded-oblong, orbicular, less than 0.5 cm long; main vein simple, occasionally obsolete near apex; sori solitary on the main vein	***D.motleyi***
–	Fronds longer than 1 cm, oblong, oblong-ovate; sori many	**5**
5	Fronds oblong, oblong-ovate, broadest at lower part; sterile frond not lobed	***D.sublimbatum***
–	Fronds oblong, spatulate; broadest at upper part; sterile frond occasionally lobed	***D.henzaianum***
6	Fronds round to elliptic; sori usually crowded in the apical part of fronds	***D.bimarginatum***
–	Fronds oblong-spatulate, lobed distinct; sori born at ultimate segments of lobe	***D.mindorense***

## Supplementary Material

XML Treatment for Didymoglossum radiatum

XML Treatment for Didymoglossum henzaianum

XML Treatment for Didymoglossum mindorense

## ﻿Additional information

### Use of AI

No use of AI was reported.

### Author ORCIDs

Siwakorn Chokrassameehirun https://orcid.org/0009-0007-3194-4724

Ekaphan Kraichak https://orcid.org/0000-0002-8437-2180

Tassanai Jaruwattanaphan https://orcid.org/0000-0002-5056-1039

### Data availability

All of the data that support the findings of this study are available in the main text.

## ﻿Appendix I

**Table A1.** Voucher and GenBank accession numbers of *rbc*L sequences used in this study.

Taxa	Voucher and locality	GenBank accession number	References
*D.angustifrons*	*J.H. Nitta 120* (UC, CR, TI), Costa Rica	MW138292	Nitta et al. 2020
*D.ballardianum*	*Droissart Kamdem 2293* (BRLU, YA), Cameroon	OQ162193	Dubuisson et al. 2023
*D.barklyanum*	*E. Grangaud s.n.* (pers. herb.), Reunion	KF992473	Hennequin et al. 2014
*D.beaverianum*	*B. Senterre 6577* (P), Seychelles	KY039369	Senterre et al. 2017
*D.beccarianum*	*B. Senterre 5899* (P), Seychelles	KY039365	Senterre et al. 2017
*D.beccarianum*	*B. Senterre 6415* (P), Seychelles	KY039367	Senterre et al. 2017
*D.benlii*	*Norris 105848* (UC), Equatorial Guinea	OQ162198	Dubuisson et al. 2023
*D.bimarginatum*	TNS:777865, Taiwan	AB574713	Ebihara et al. 2010
*D.bimarginatum*	*K.R. Wood 14098* (PTBG), Micronesia	MT657805	Nitta et al. 2020 (unpublished)
*D.bimarginatum*	*K.R. Wood 16940* (PTBG), Samoa	MT657806	Nitta et al. 2020 (unpublished)
*D.bimarginatum*	*S. Chokrassameehirun 273*, Thailand	PQ605414	this study
*D.bimarginatum*	*S. Chokrassameehirun 142*, Thailand	PQ605415	this study
D.sp.cf.kapplerianum	*JN-2020 J.H. Nitta* (UC, CR, TI), Costa Rica	MW138281	Nitta et al. 2020
*D.chamaedrys*	*Senterre 7025* (BRLU), Liberia	OQ162203	Dubuisson et al. 2023
*D.erosum*	*Bidault 3433* (BRLU), Gabon	OQ162194	Dubuisson et al. 2023
*D.exiguum*	*Iwatsuki 99H09* (TNS), Thailand	AB257488	Ebihara et al. 2007
*D.exiguum*	*S. Chokrassameehirun 257*, Thailand	PQ605413	this study
*D.henzaianum*	*S. Chokrassameehirun 22*, Thailand	PQ605408	this study
*D.henzaianum*	*S. Chokrassameehirun 101*, Thailand	PQ605419	this study
*D.kapplerianum*	*J.H. Nitta 2299A* (UC, GH), Costa Rica	MW138136	Nitta et al. 2020
*D.kirkii*	*Rouhan 925* (P), Comoros	OQ162185	Dubuisson et al. 2023
*D.krausii*	*Chaussidon 43-2007* (P), Martinique	OQ162176	Dubuisson et al. 2023
*D.lenormandii*	*Rakotondrainibe 6260* (P), Madagascar	OQ162188	Dubuisson et al. 2023
*D.liberiense*	*Rakontodrainibe 1880* (P), Madagascar	OQ162182	Dubuisson et al. 2023
*D.lorencei*	*J.-Y. Dubuisson HR 2002-7* (P, REU), Reunion	KF992474	Hennequin et al. 2014
*D.membranaceum*	*Chaussidon 14-2007* (P), Guadeloupe	OQ162187	Dubuisson et al. 2023
*D.mindorense*	*Ebihara 010909-01* (TNS), Australia	OQ162186	Dubuisson et al. 2023
*D.mindorense*	*S. Chokrassameehirun 228*, Thailand	PQ605412	this study
*D.motleyi*	TNS:759342, Japan	AB574714	Ebihara et al. 2010
*D.motleyi*	*S. Chokrassameehirun 102*, Thailand	PQ605411	this study
*D.petersii*	*FLAS 261024*, GA, Coffee Co.	MF987839	Pinson et al. 2017
*D.punctatum*	*Chaussidon s.n.* (P), Martinique	OQ162178	Dubuisson et al. 2023
*D.reptans*	*Salino 13280* (BHCB), Brazil	OQ162177	Dubuisson et al. 2023
*D.rotundifolium*	*Pignal 3484* (P), Comoros	OQ162190	Dubuisson et al. 2023
D.sp. aff.tahitense	*J.Nitta 005*, French Polynesia	EU122975	Nitta 2008
*D.radiatum*	*S. Chokrassameehirun 259*, Thailand	PQ605406	this study
*D.radiatum*	*S. Chokrassameehirun 241*, Thailand	PQ605407	this study
*D.sublimbatum*	*R. Knapp 3688* (P), Taiwan	KY039371	Senterre et al. 2017
*D.sublimbatum*	*S. Chokrassameehirun 281*, Thailand	PQ605409	this study
*D.sublimbatum*	*S. Chokrassameehirun 230*, Thailand	PQ605410	this study
*D.sublimbatum*	*S. Chokrassameehirun 04*, Thailand	PQ605420	this study
*D.tahitense*	*Schuettpelz 1864* (US), French Polynesia	MT216014	Steier and Schuettpelz 2020 (unpublished)
*D.tahitense*	*K.R. Wood 13651* (PTBG), Micronesia	MT657808	Nitta et al. 2020 (unpublished)
*D.tahitense*	*K.R. Wood 16865* (PTBG), Samoa	MT657809	Nitta et al. 2020 (unpublished)
*T.tahitense*	*Ebihara 001119-01* (TNS), Japan	AB257498	Ebihara et al. 2007
*C.minutum*	*S. Chokrassameehirun 283*, Thailand	PQ605416	this study
*H.acanthoides*	*S. Chokrassameehirun 74*, Thailand	PQ605418	this study
*H.baileyanum*	*H. Streimann s.n.* (UC), Australia	AF275643	Pryer et al. 2001
